# Use of T2-weighted magnetic resonance imaging of the optic nerve sheath to detect raised intracranial pressure

**DOI:** 10.1186/cc7006

**Published:** 2008-09-11

**Authors:** Thomas Geeraerts, Virginia FJ Newcombe, Jonathan P Coles, Maria Giulia Abate, Iain E Perkes, Peter JA Hutchinson, Jo G Outtrim, Dot A Chatfield, David K Menon

**Affiliations:** 1 University Division of Anaesthesia and Wolfson Brain Imaging Center, University of Cambridge, Addenbrooke's Hospital, Hills Road, Cambridge, CB2 2QQ, UK; 2 Département d'Anesthésie-Réanimation Chirurgicale, AP-HP and University Paris-Sud, Centre Hospitalier Universitaire Bicêtre, rue du General Leclerc, Le Kremlin Bicêtre, 94275, France; 3 Department of Neurosurgery and Wolfson Brain Imaging Center, University of Cambridge, Addenbrooke's Hospital, Hills Road, Cambridge, CB2 2QQ, UK

## Abstract

**Introduction:**

The dural sheath surrounding the optic nerve communicates with the subarachnoid space, and distends when intracranial pressure is elevated. Magnetic resonance imaging (MRI) is often performed in patients at risk for raised intracranial pressure (ICP) and can be used to measure precisely the diameter of optic nerve and its sheath. The objective of this study was to assess the relationship between optic nerve sheath diameter (ONSD), as measured using MRI, and ICP.

**Methods:**

We conducted a retrospective blinded analysis of brain MRI images in a prospective cohort of 38 patients requiring ICP monitoring after severe traumatic brain injury (TBI), and in 36 healthy volunteers. ONSD was measured on T2-weighted turbo spin-echo fat-suppressed sequence obtained at 3 Tesla MRI. ICP was measured invasively during the MRI scan via a parenchymal sensor in the TBI patients.

**Results:**

Measurement of ONSD was possible in 95% of cases. The ONSD was significantly greater in TBI patients with raised ICP (>20 mmHg; 6.31 ± 0.50 mm, 19 measures) than in those with ICP of 20 mmHg or less (5.29 ± 0.48 mm, 26 measures; *P *< 0.0001) or in healthy volunteers (5.08 ± 0.52 mm; *P *< 0.0001). There was a significant relationship between ONSD and ICP (*r *= 0.71, *P *< 0.0001). Enlarged ONSD was a robust predictor of raised ICP (area under the receiver operating characteristic curve = 0.94), with a best cut-off of 5.82 mm, corresponding to a negative predictive value of 92%, and to a value of 100% when ONSD was less than 5.30 mm.

**Conclusions:**

When brain MRI is indicated, ONSD measurement on images obtained using routine sequences can provide a quantitative estimate of the likelihood of significant intracranial hypertension.

## Introduction

Raised intracranial pressure (ICP) is frequent in conditions such as stroke, liver failure, meningitis, meningoencephalitis and postresuscitation syndrome [1-6]. In such diseases, raised ICP may be associated with increased mortality and poor neurological outcomes as a result of ischaemic insults to the brain [4,7]. Early detection and treatment of raised ICP is therefore critical but often challenging, because invasive ICP monitoring is not routinely undertaken in these settings. However, magnetic resonance imaging (MRI) is often undertaken in such patients, and may provide a noninvasive method of estimating ICP. The optic nerve, as a part of the central nervous system, is surrounded by a subarachnoid space and experiences the same pressure changes as the intracranial compartment [8-11]. The intraorbital part of the sheath, and particularly its retrobulbar segment, can distend when ICP (and hence cerebrospinal fluid [CSF] pressure) is elevated. MRI can be used to measure precisely the diameter optic nerve and its surrounding sheath, by using a fat-suppressed T2-weighted sequence [12,13]. In cases of idiopathic intracranial hypertension or papilloedema, the retrobulbar optic nerve sheath diameter (ONSD), measured using MRI, has been reported to be enlarged [14]. Moreover, in cases of hypotension in the CSF, ONSD was found to be reduced [15]. However, the precise correlation between MRI-determined ONSD and invasive ICP, which remains the 'gold standard' for ICP measurement, has never been studied. Such a comparison is essential to calibrate the MRI estimation of ICP and to define thresholds for diagnosing intracranial hypertension.

In our institution, brain MRI studies are performed for research purposes during the acute phase of traumatic brain injury (TBI), with a substantial proportion performed in sedated and mechanically ventilated patients with invasive ICP monitoring. We therefore undertook a retrospective analysis of MRI scans in a cohort of TBI patients with ICP monitoring in order to study the relationship between ONSD and ICP and to assess the diagnosis accuracy of ONSD for the detection of raised ICP. MRI scans obtained from healthy volunteers were also studied to define normal values for optic nerve and ONSD.

## Materials and methods

### Patients

This study was a retrospective analysis of data collected prospectively between October 2006 and April 2008 from severe TBI patients with postresuscitation Glasgow Coma Scale score of 8 or less, who required sedation, mechanical ventilation and ICP monitoring. All patients were treated with protocol driven therapy aimed to maintain ICP below 20 mm Hg and cerebral perfusion pressure between 60 and 70 mmHg [16]. Patients were sedated using intravenous propofol and fentanyl, and were mechanically ventilated. Patients were eligible for inclusion if they had undergone MRI, including a T2-weighted sequence, within 1 week of injury. Injury Severity Score and Simplified Acute Physiology Score II were calculated from values obtained at day 1 after patient arrival [17,18]. Next of kin assent was obtained in all cases. Ethical approval was obtained from the local research ethics committee. Age-matched control individuals (healthy volunteers recruited from the local community by advertisement) who underwent an identical imaging protocol during the same period were also studied. Exclusion criteria for healthy volunteers included a history of psychiatric or physical illness (particularly cardiovascular or neurological disorders), head injury and any history of drug or alcohol dependence, as well as contraindications for MRI.

### Intracranial monitoring

ICP was continuously measured in TBI patients via an intraparenchymal probe (Codman & Shurlett Inc., Ryanham, MA, USA) inserted into the frontal lobe via Technicam Cranial Access^® ^device (Technicam Ltd, Newton Abbot, UK) by a neurosurgeon. During MRI, the ICP transducer and excess wire were located outside the receive head coil, as has previously been described to be safe and not to cause heating [19]. ICP was continuously monitored during MRI, and readings were collected on a monitoring chart every 10 minutes. The ICP value corresponding to the exact time of acquisition of the T2-weighted MRI sequence was recorded. Raised ICP was defined as ICP above 20 mmHg.

### Imaging protocol

**Figure 1:**
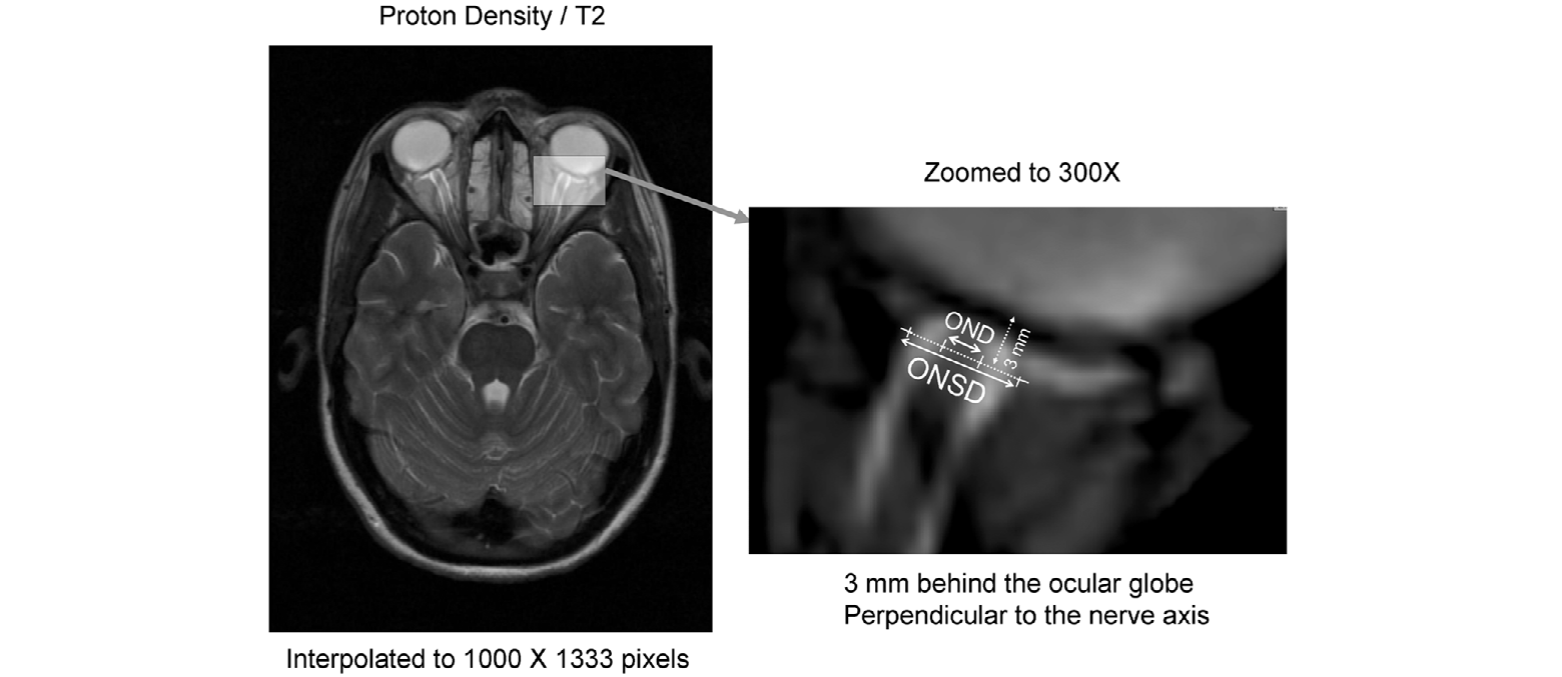
Methodology to measure ONSD and OND. OND, optic nerve diameter; ONSD, optic nerve sheath diameter.

MRI in all individuals was performed using a 3T Magnetom Total Imaging Matrix Trio (Siemens Medical Solutions, Munich, Germany). The axial proton density/T2-weighted turbo spin-echo fat-suppressed sequence was used to measure ONSD and optic nerve diameter (OND). The scan parameters were as follows: repetition time 4,600 ms, echo time 12 ms, pixel bandwidth 185 Hz/pixel, slice thickness 4 mm, spacing between slices 5 mm, and number of slices 27. The optic nerve sheath appeared as a high signal surrounding a region of low signal corresponding to the optic nerve (Figure 1). The axial image slice that provided the best view of the ONSD was chosen and the slice was interpolated to 1,000 × 1,333 pixels using Image J 1.38 (National Institutes of Heath, Bethesda, MD, USA). The retrobulbar area was zoomed to 300×, and then ONSD and OND were measured in an axis perpendicular to the optic nerve, 3 mm behind the globe using an electronic caliper. The OND and the ONSD values obtained from both sides were averaged for comparison with ICP measurements.

Computed tomography (CT) classification was determined from the head CT scan obtained on admission. CT findings were classified as described by Marshall and coworkers [20]: diffuse injury (DI) category I corresponds to no visible intracranial damage on CT scan; DI category II corresponds to a midline shift of 0 to 5 mm; DI category III corresponds to absent or compressed cisterns, with a midline shift of 0 to 5 mm; DI category IV corresponds to a midline shift of more than 5 mm; evacuated mass lesion (EML) corresponds to any surgically evacuated lesion; and nonevacuated mass lesion (NEML) corresponds to a high-density or mixed-density lesion more than 25 mm in diameter and not surgically evacuated.

### Statistical analysis

Statistical analyses were conducted using Statview (Statview 5.0 software; SAS Institute Inc., Cary, NC, USA). After assessment for normality, parametric comparisons were performed using two-tailed Student's *t*-test. Proportions were compared using χ^2 ^test. To evaluate the diagnostic accuracy for raised ICP, receiver operating characteristic (ROC) curves were produced for ONSD and OND using Medcalc 9.1 Software (Frank Schoonjans, Mariakerke, Belgium). Values are expressed as means ± standard deviation otherwise specified, and *P *values < 0.05 were considered to be statistically significant.

### Role of funding sources

The sponsors of this study and the funding sources played no role in the study design, data collection, data analysis, data interpretation, or writing of the report.

## Results

### Study population

Thirty-eight TBI patients were studied. Seven of them had two MRI studies during the acute phase of TBI, resulting in 45 scans with ICP monitoring. ONSD and OND measurements were possible for both sides in 97% in TBI patients (only left-sided measurements were possible in one patiennt). Thirty-seven healthy volunteers were initially studied. One was excluded because of lack of adequate view of both optic nerves. In two other cases, ONSD and OND were only measured on one side. The overall feasibility of measuring ONSD and OND was therefore calculated as 95% (78/82 scans corresponding to 72/75 individuals). Demographic characteristics of the population are presented in Table 1.

**Table 1:** General and intracranial characteristics of patients and healthy volunteers.

Characteristics	Brain-injured patients	Healthy volunteers	*P *value
	
Number of patients	38	36	-
Number of scans	45	36	-
Age (years)	35 ± 14	32 ± 8	0.37
Weight (kg)	86 ± 14	75 ± 10	0.0003
Sex (% male)	80%	75%	0.82
Overall ICU length of stay (days)	17 ± 9	-	-
SAPS II score	21 ± 6	-	-
Injury Severity Score (median [range])	25 (9–50)	-	-
Glasgow Coma Score (median [range])	7 (3–8)	-	-
Intracranial pressure (mmHg; [range])	18.7 ± 5.6 (7–34)	-	-
Optic nerve sheath diameter (mm)	5.72 ± 0.71	5.08 ± 0.52	0.0001
Optic nerve diameter (mm)	2.65 ± 0.28	2.70 ± 0.23	0.26
Head CT scan (Marshall's category; % of patients)			
DI category I	0%	-	-
DI category II	55%	-	-
DI category III	3%	-	-
DI category IV	0%	-	-
EML	39%	-	-
NEML	3%	-	-

Unless otherwise stated, values are expressed as mean ± standard deviation. DI, diffuse injury; EML, evacuated mass lesion; ICU, intensive care unit; NEML, nonevacuated mass lesion; SAPS, Simplified Acute Physiology Score.

### Optic nerve sheath and optic nerve diameters

The mean ONSD in the TBI population was 5.72 ± 0.71 mm, ranging from 4.38 to 7.25 mm. The mean ONSD in healthy volunteers was significantly lower (5.08 ± 0.52 mm; *P *= 0.0001). The ONSD was significantly higher in TBI patients with raised ICP (>20 mmHg; 6.31 ± 0.50 mm, n = 19) than in TBI patients with ICP of ≤ 20 mmHg (5.29 ± 0.48 mm, n = 26; *P *< 0.0001) and in healthy volunteers (*P *< 0.0001). ONSD in TBI patients with ICP of ≤ 20 mmHg and in healthy volunteers were not significantly different (*P *= 0.12).

The mean OND in the TBI population was 2.65 ± 0.28 mm, ranging from 2.12 to 3.27 mm, and was not significantly different from that in healthy volunteers (2.70 ± 0.23 mm; *P *= 0.26). OND did not differ between the TBI patients with raised ICP (2.74 ± 0.23 mm) and those with normal ICP (2.58 ± 0.29 mm; *P *= 0.10) and healthy volunteers (*P *= 0.71).

### Relationship between optic nerve sheath diameter, optic nerve diameter and intracranial pressure

**Figure 2:**
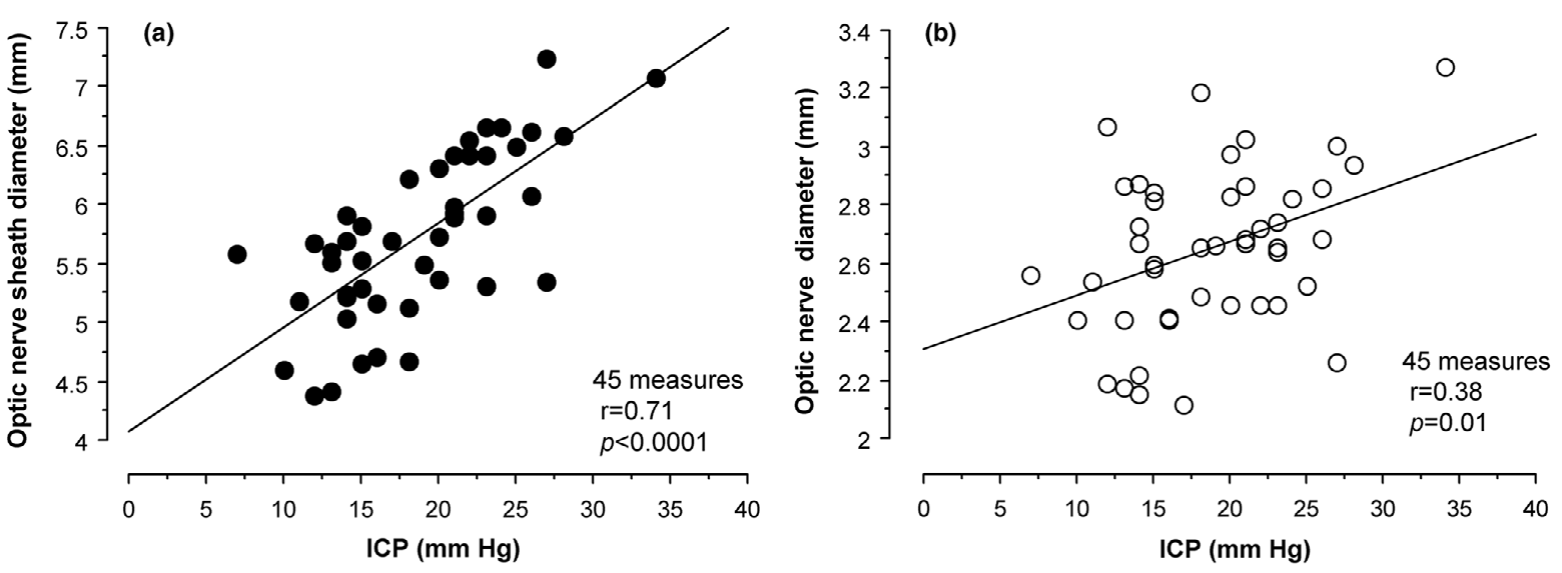
Parenchymal ICP versus ONSD and OND. Presented is the relationship between parenchymal ICP and **(a) **ONSD and **(b) **OND. Linear regression analysis identified a strong and significant relationship between ICP and ONSD. ICP, intracranial pressure; OND, optic nerve diameter; ONSD, optic nerve sheath diameter.

The mean ICP in TBI patients was 18.7 ± 5.7 mmHg, ranging from 7 to 34 mmHg. A significant and strong linear relationship was found between ONSD and ICP (*r *= 0.71, *P *< 0.0001; Figure 2a). The 95% confidence limit for the prediction of ICP using ONSD was 9 mmHg. A weaker relationship was found between OND and ICP (*r *= 0.38, *P *= 0.01; Figure 2b).

### Optic nerve sheath diameter to detect raised intracranial pressure

**Figure 3:**
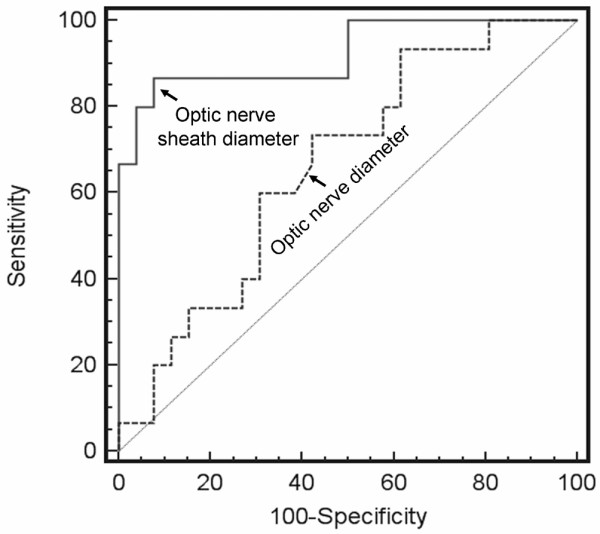
ROC curves for ONSD and OND with respect to raised ICP. 'Raised ICP' is defined as an ICP above 20 mmHg identified during T2-weighted magnetic resonance imaging. ICP, intracranial pressure; OND, optic nerve diameter; ONSD, optic nerve sheath diameter; ROC, receiver operating characteristic.

ONSD accurately predicted an ICP greater than 20 mmHg (area under ROC curve = 0.94, 95% confidence interval = 0.86 to 1.01; *P *= 0.0001; Figure 3). The best cut-off value of ONSD for detecting raised ICP was 5.82 mm, with a sensitivity of 90%, a specificity of 92% and a negative predictive value of 92%. One hundred per cent sensitivity and negative predictive values were achieved for a 5.30 mm ONSD cut-off. However, OND did not accurately predict raised ICP (area under ROC curve = 0.68, 95% confidence interval = 0.53 to 0.84; *P *= 0.05). These two ROC curves were significantly different (*P *= 0.001).

### Inter-observer variability

**Figure 4:**
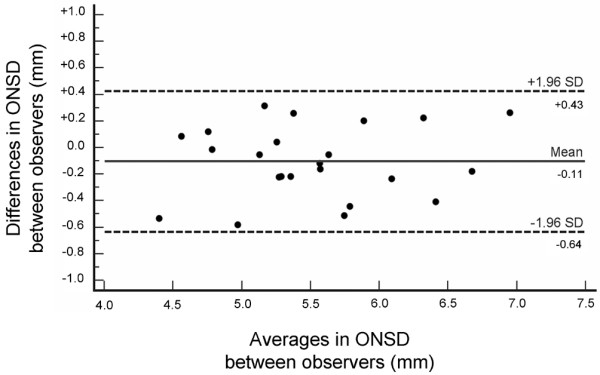
Agreement between observers in measurement of ONSD. Presented is a Bland-Altman [37] graphical representation. ONSD, optic nerve sheath diameter; SD, standard deviation.

The inter-observer variability in OND and ONSD measurement was assessed by comparing measurements of ONSD and OND in TBI and healthy volunteers. The observers (TG and VN) were blinded to each other's findings, to ICP and to subjects' identity. The inter-observer variability was tested on 23 randomly selected MRI datasets, corresponding to 22 individuals (12 healthy volunteers and 10 TBI patients). The mean standard deviation for ONSD was 0.17 mm, and the mean difference between observers for ONSD was 0.11 mm (Table 2). The agreement between observers is presented in Figure 4.

**Table 2:** Inter-observer variability for optic nerve (and sheath) diameters

	Optic nerve diameter	Optic nerve sheath diameter
Mean standard deviation (mm)	0.25	0.17
Mean difference (mm)	0.30	0.11

The inter-observer variability was tested in 23 randomly selected magnetic resonance imaging datasets.

## Discussion

This study shows that MRI using ONSD measurement is potentially useful in detecting raised ICP. ONSD (but not OND) is strongly related to ICP, a finding that reflects distension of the nerve sheath during increases in CSF pressure. The negative predictive value of an ONSD under 5.82 mm for having ICP above 20 mmHg is more than 90%.

The early detection of raised ICP can be very difficult when invasive devices are not available. Clinical signs of raised ICP such as headache, vomiting and drowsiness are not specific and often difficult to interpret. In sedated patients, clinical signs of raised ICP frequently appear late, when ischaemic brain injury is already established [21]. Furthermore, a normal CT scan does not exclude a raised ICP [22-24].

Fundoscopic evidence of papilloedema can provide useful evidence of intracranial hypertension in cases of chronic raised ICP [25]. However, experimental studies clearly show that oedema of the optic disk requires a few days to develop and resolve, making it a less useful clinical sign in settings where there may be acute increases or dynamic changes in ICP [26]. However, papilloedama is a delayed consequence of chronic CSF accumulation in the retrobulbar optic nerve dural sheath due to raised pressure in CSF in cranial cavity, and direct measurement of such CSF accumulation may provide an earlier and more responsive measure of intracranial hypertension. Optic nerve sheath distension could therefore be an earlier, more reactive and more sensitive sign of raised ICP.

High-resolution MRI is accurate at measuring ONSD [27,28] and has been proposed to detect raised ICP in idiopathic hydrocephalus and to diagnose shunt malfunction [12,14,29,30]. On T2-weighted sequences, water (and CSF) exhibits a high signal (white). Fat and grey matter appear as light grey, and white matter as dark grey. The perioptic CSF is surrounded by orbital fat. Contrast between CSF and orbital fat can be improved with fat suppression, increasing the image resolution for the ONSD measurement [12,13]. We have confirmed the utility of this approach, and we provide – for the first time – a quantitative estimate of the relationship between MRI-determined ONSD and ICP. Perhaps more importantly, our data also provide predictive thresholds that enable the exclusion of significant intracranial hypertension. In this context, MRI-derived measurement of ONSD has a low inter-observer variability (less than 0.2 mm), which is substantially less than the mean difference in the measurement between raised ICP and normal ICP patients (1.02 mm) and healthy volunteers (1.23 mm). As a screening test, the technique has a high sensitivity and negative predictive value. For an ONSD cut-off of 5.82 mm, the sensitivity and negative predictive value were 90% and 92%, respectively. A lower cut-off of 5.30 mm resulted in a sensitivity and negative predictive value of 100% for a diagnosis of significant intracranial hypertension, but at the cost of a reduction in specificity to 50%. The most useful clinical message derived from our data may be the following thresholds; an ONSD less than 5.30 mm is unlikely to be associated with raised ICP, and an ONSD above 5.82 mm indicates that the probability of raised ICP is 90%. However, OND had a weaker positive relationship with ICP. Therefore, the increase in ONSD during raised ICP cannot be related only to optic nerve dilatation (as during optic nerve oedema) but also, and predominantly, to its optic nerve sheath distension, probably caused by increased CSF pressure around the optic nerve.

In the present study, TBI patients have significantly greater weights than healthy volunteers (*P *= 0.0003). Because optic nerve diameters may be related to body size, this could induce a significant bias. However, body weights in TBI patients with raised ICP (n = 14) were not significantly different from those without raised ICP (n = 24; respectively, 89.0 ± 15.8 kg and 84.1 ± 12.6 kg; *P *= 0.3). The differences observed in ONSD between TBI patients with and without raised ICP can therefore not be attributed to differences in body weight.

A major limitation of this study is probably related to MRI by itself, with limited access, necessity of patient transfer in a magnetic area and specific contraindications. Moreover, in the present study, the slice thickness and interslice spacing were relatively large (4 and 5 mm, respectively). The optic nerve and its sheath were therefore apparent in only one or two slices of the T2-weighted turbo spin-echo sequence. MRI protocols using thinner slices or three-dimensional volumetric acquisition could probably increase the precision of the measurement of ONSD. Consequently, this test should not be used to predict the exact value of ICP but to estimate the probability of intracranial hypertension.

Developing a reliable measurement of ONSD is of interest. In humans, noninvasive assessment of ICP using ocular sonography and ONSD measurement has been proposed in cases of hydrocephalus, hepatic failure and TBI [8,10,31-36]. Interestingly, the best cut-off value for raised ICP using ocular sonography was 5.7 to 5.8 mm, which is very close to the figure identified in the present study (5.8 mm). This study corroborates findings obtained with ocular sonography and provides evidence for the use of ONSD measurement when brain MRI is performed in situations that potentially can lead to raised ICP. Proton density/T2-weighted turbo spin-echo is a conventional sequence, lasting less than 3 minutes, which forms part of most routine clinical MRI studies. ONSD measurement could provide important clinical information on the likelihood of intracranial hypertension, and it may help to identify those patients who require more invasive monitoring.

## Conclusion

In sedated TBI patients, ONSD measured using conventional brain T2-weighted MRI strongly correlates with invasive ICP. An ONSD above 5.82 mm is associated with a 90% probability of significant intracranial hypertension, whereas the probability of not having significant intracranial hypertension is 90% if the ONSD is under 5.82 mm and 100% if it is less than 5.30 mm. When MRI is indicated, ONSD can easily be measured on routine sequences, and provides a quantitative estimate of the likelihood of significant intracranial hypertension.

## Key messages

• The dural sheath surrounding the optic nerve communicates with the subarachnoid space and distends when intracranial pressure is elevated.

• MRI can be used to precisely measure the diameter of optic nerve and its sheath.

• The ONSD is significantly greater in TBI patients with raised ICP (>20 mmHg) than in those with ICP ≤ 20 mmHg or healthy volunteers. Enlarged ONSD is a robust predictor of raised ICP (area under ROC curve = 0.94).

• An ONSD less than 5.30 mm is unlikely to be associated with raised ICP, and an ONSD above 5.82 mm is associated with a 90% probability of raised ICP.

• MRI-derived measurement of ONSD has low inter-observer variability.

## Abbreviations

CSF: cerebrospinal fluid; CT: computed tomography; DI: diffuse injury; ICP: intracranial pressure; MRI: magnetic resonance imaging; OND: optic nerve diameter; ONSD: optic nerve sheath diameter; ROC: receiver operating characteristic; TBI: traumatic brain injury.

## Competing interests

The authors declare that they have no competing interests.

## Authors' contributions

TG conceived of the study, collected data, performed statistical analysis and drafted the manuscript. VFJN collected data and helped to perform statistical analysis and draft the manuscript. JPC participated in study design and helped to draft the manuscript. MGA, IEP, JGO and DAC helped to collect data. PJAH helped to draft the manuscript. DKM participated in the study design and coordination, and helped to draft the manuscript. All authors read and approved the final manuscript.
